# Epidemiology and Clinical Characteristics of Human Rickettsial Infections Detected in Nigeria: Discovery of a Significant Contributor to Acute Febrile Illness

**DOI:** 10.1093/cid/ciaf502

**Published:** 2025-11-20

**Authors:** Vivian Gga Kwaghe, Cyril Erameh, Lauren P Courtney, Jay Osi Samuels, Claire A Quiner, Jean H Kim, Osahogie Isaac Edeawe, Nankpah Godsave Vongdip, Adamu Zigwai Ephraim, Onyia Justus Ejike, Ikponmwosa Odia, Kat Asman, Philippe Chebu, Jacqueline Agbukor, Oladimeji Damilare Matthew, Victoria Orok, Femi Owolagba, Blessed Okhiria, Ephraim Ogbaini-Emovon, Walter Mary Odion, Blessing Amierhobhiye Obagho, Richard Fayomade, Emmanuel A Oga

**Affiliations:** University of Abuja Teaching Hospital, Gwagwalada, Federal Capital Territory, Nigeria; Irrua Specialist Teaching Hospital, Edo, Nigeria; Solutions, RTI International, Durham, North Carolina; APIN Public Health Initiatives, Federal Capital Territory, Nigeria; Solutions, RTI International, Durham, North Carolina; Solutions, RTI International, Durham, North Carolina; Irrua Specialist Teaching Hospital, Edo, Nigeria; University of Abuja Teaching Hospital, Gwagwalada, Federal Capital Territory, Nigeria; Solutions, RTI International, Durham, North Carolina; University of Abuja Teaching Hospital, Gwagwalada, Federal Capital Territory, Nigeria; Irrua Specialist Teaching Hospital, Edo, Nigeria; Solutions, RTI International, Durham, North Carolina; APIN Public Health Initiatives, Federal Capital Territory, Nigeria; Irrua Specialist Teaching Hospital, Edo, Nigeria; University of Abuja Teaching Hospital, Gwagwalada, Federal Capital Territory, Nigeria; University of Abuja Teaching Hospital, Gwagwalada, Federal Capital Territory, Nigeria; APIN Public Health Initiatives, Federal Capital Territory, Nigeria; University of Abuja Teaching Hospital, Gwagwalada, Federal Capital Territory, Nigeria; Irrua Specialist Teaching Hospital, Edo, Nigeria; Irrua Specialist Teaching Hospital, Edo, Nigeria; Irrua Specialist Teaching Hospital, Edo, Nigeria; APIN Public Health Initiatives, Federal Capital Territory, Nigeria; University of Abuja Teaching Hospital, Gwagwalada, Federal Capital Territory, Nigeria; ClineEpi Partners, Columbia, Maryland

**Keywords:** Human rickettsial infection, Nigeria

## Abstract

**Introduction:**

Rickettsial diseases are transmitted by arthropods and characterized by nonspecific febrile illness. Despite evidence of *Rickettsia* spp. in animals and vectors in Nigeria, human infection has not previously been documented to the best of our knowledge. Most clinicians lack awareness about these diseases. The burden of rickettsiosis in Nigeria is unknown.

**Methods:**

The Surveillance of Acute Febrile Illness Aetiologiesin Nigeria study was a facility-based surveillance study designed to identify pathogens causing acute fever. Whole blood specimens from patients with undifferentiated fever were tested using a TaqMan Array Card, a polymerase chain reaction assay including *Rickettsia* spp., and an enzyme-linked immunosorbent assay for *Rickettsia* spp. immunoglobulin G and immunoglobulin M. Demographic, clinical, and outcome data were collected and analyzed.

**Results:**

Of the 1200 patients enrolled, 26% tested positive for *Rickettsia* spp., via TaqMan Array Card and 28.7% via enzyme-linked immunosorbent assay. Common clinical characteristics were headache, nausea and vomiting, abdominal pain, muscle pain, and joint pain. Only 1.9% of the patients had a rash and none had an eschar. The overall case fatality rate was 4.2%, with significantly lower mortality in children compared to adults (0.8% vs 6.5%). Coinfections were detected in 36.2% of cases, most commonly with *Plasmodium* spp. (25%).

**Conclusions:**

This study provides the first evidence of human rickettsial infection in humans in Nigeria, with a substantial prevalence among febrile patients. The absence of classic cutaneous signs such as rash or eschar may hinder clinical recognition. Rickettsial infection should be considered in the differential diagnosis of acute febrile illness in Nigeria, and molecular diagnostics should be expanded to improve detection and guide appropriate therapy.

## Teaser

This study reveals the first reported human rickettsial infections in Nigeria, highlights diagnostic challenges resulting from absent skin symptoms, and urges inclusion in febrile illness diagnoses and expansion of molecular testing for accurate detection and treatment.

Rickettsial infections are a group of zoonotic diseases caused by obligate intracellular, gram-negative bacteria of the order *Rickettsiales*, including the genera *Rickettsia*, *Orientia*, *Anaplasma*, and *Ehrlichia* [1, 2]. These pathogens are transmitted to humans primarily through arthropod vectors such as ticks, fleas, lice, and mites. The genus *Rickettsia* is further classified into the spotted fever group (SFG), typhus group, transitional group, and ancestral group [2]. The SFG, the largest subgroup, includes *Rickettsia rickettsii*, *Rickettsia conorii*, and *Rickettsia africae*, which are primarily tick-borne and associated with significant morbidity and mortality worldwide [3].

Rickettsial diseases are reemerging zoonotic diseases that are prevalent globally. The infections are transmitted to human hosts mostly through arthropod bites or arthropod feces that infect scratching lesions. They are not transmissible directly from person to person except by blood transfusion or organ transplantation. The causative species and epidemiology vary depending on the region of the world; *R.rickettsii* causes Rocky Mountain spotted fever, the most severe and most well-known of the rickettsial infections in North America, whereas *R. africae* causes African tick bite fever in sub-Saharan Africa, and *R. conorii* causes Mediterranean spotted fever in Europe and North Africa [4].

In sub-Saharan Africa, where malaria is endemic, undifferentiated febrile illnesses are attributed to malaria and as such, surveillance of rickettsial diseases, among others, is limited or nonexistent. In travelers returning from Africa, rickettsiosis was previously reported as the second most frequent cause of fever after malaria [5].

Vasculitis is the basic pathogenetic mechanism of rickettsial diseases, which leads to disseminated inflammation and increased vascular permeability throughout the body [6]. Infection of the endothelial cells also induces procoagulant activity that promotes coagulation factor consumption, platelet adhesion, and leukocyte emigration and may result in a clinical syndrome similar to disseminated intravascular coagulation [7].

Rickettsial diseases present as an acute undifferentiated febrile illness, associated with headaches, myalgia, and malaise. Cutaneous manifestations include rash and eschar [8]. Acute complications include pneumonitis, acute respiratory distress syndrome, meningoencephalitis, jaundice, acute kidney injury, myocarditis, and septic shock [9]. The disease can be life threatening if not treated with appropriate antibiotic therapy early in the infection.

Although human rickettsioses have not been previously documented in Nigeria, evidence from veterinary and entomologic studies indicates that pathogenic *Rickettsia* species are present in the country. Molecular surveys have identified *R. massiliae* in Rhipicephalus ticks from small ruminants [10] and *R. africae* in *Amblyomma variegatum* ticks infesting livestock [11]. These findings suggest ongoing transmission cycles involving humans, animals, and vectors.

The Surveillance for Acute Febrile Illness Aetiology in Nigeria (SAFIAN) study is a facility-based surveillance study that was conducted in Nigeria to identify fever-causing pathogens. This study was conducted at 2 tertiary hospitals. A total of 1200 clinical specimens from acutely febrile patients were screened for 25 pathogens, including *Rickettsia* spp. using Thermo Fisher's TaqMan Array Cards (TAC). As a convenience sample, specimens from 1 site were also tested using enzyme-linked immunosorbent assay assays for *Rickettsia* spp. Immunoglobulin G (IgG) and IgM. This paper describes the first reported detection of *Rickettsia* spp. in humans in Nigeria, as well as its prevalence, clinical characteristics, and outcomes as identified by the SAFIAN study.

## METHODOLOGY

### Study Sites

A comprehensive description of the methods, procedures, and details of the SAFIAN study can be found in detail in Courtney et al (2025). In brief, the study was conducted at 2 tertiary hospitals in Nigeria: University of Abuja Teaching Hospital (UATH) located in Gwagwalada, a semiurban local government area in the Federal Capital Territory (FCT), and Irrua Specialist Teaching Hospital (ISTH) located in Irrua, a rural settlement in Edo State. FCT and Edo State are situated in the north central and the south zones of Nigeria, respectively. UATH is a 520-bed hospital and ISTH is a 350-bed hospital.

### Study Design

This was a facility-based active surveillance study designed to detect up to 25 acute febrile illness (AFI)-causing pathogens among study participants from September 2023 to August 2024.

### Study Population

We enrolled 1200 adults and children with acute fever within the past 10 days who presented to UATH or ISTH seeking medical care. Participants were screened for eligibility and enrolled in the study within 24 hours of presenting at the hospital if they fulfilled the eligibility criteria. To capture seasonal variation of pathogens, participants were enrolled over a 12-month period, September 2023 to August 2024, with equal enrollment each month.

### Study Methods

A comprehensive description of the methods and procedures can be found in Courtney et al, 2025. Eligible patients were consented/assented and enrolled in exchange for a ₦4000 Naira incentive. A participant survey was conducted on an electronic device (Research Electronic Data Capture). The survey obtained information on the participants' demographics, history of their present illness, medical history, travel history, and participant's recall of exposure to animals and wildlife. After patient discharge, hospital electronic medical records were obtained to collect medical data entered into Research Electronic Data Capture forms.

Whole blood (6 mL) was collected from each participant into ethylenediaminetetraacetic acid and serum tubes. All specimens were analyzed using ThermoFisher's TAC, a research-use only assay that allows for the screening of up to 25 pathogens. As a convenience sample, specimens from UATH participants were also serologically tested for *R. conorii* IgG and IgM using Vircell (Granada, Spain) following the manufacturer's protocols. Vircell's tests detect *R. conorii*, but may also identify other closely related species within the spotted fever group, such as *R. africae* and *R. rickettsii*.

### Data Analysis

The behaviors, risk factors, and characteristics of the population are described using frequencies and percentages for categorical variables, means, and standard deviations for continuous variables. Chi-square or *t*-tests were used to examine differences by age (youth and adult). Analyses were performed using SAS 9.4 (Cary, North Carolina). Data integration was conducted using R Studio 2023.06.0 (Boston, Massachusetts).

## RESULTS

### Cohort Characteristics

A total of 1200 participants (750 adults and 450 children) were enrolled in the SAFIAN study. Each study site enrolled 375 adults and 225 children. A total of 312 (184 adults and 128 children) participants screened positive for *Rickettsia* spp., giving an overall prevalence of 26% among enrollees with AFI. An overall difference in sex among patients with or without *Rickettsia* detection, was not observed; however, at the site level, significantly more adult males were affected in Edo State compared with those enrolled at FCT (*P* value = .012) (Table 1).

**Table 1.** Demographic Characteristics of Positive Rickettsial Patients Enrolled in SAFIAN Study by Site and Age

		Study Sites			Age		
Characteristic	Total (N = 312)	FCT (N = 161)	Edo State (N = 151)	*P*	Adult (N = 184)	Children (N = 128)	*P*
Age (mean [min, max])	30.1 (5.0–90.0)	29.5 (5.0–90.0)	30.6 (5.0–82.0)		43.0 (18.0–90.0)	11.5 (5.0–17.0)	
Gender							
Male	161 (51.6%)	72 (44.7%)	89 (58.9%)	.012	91 (49.5%)	70 (54.7%)	.363
Female	151 (48.4%)	89 (55.3%)	62 (41.1%)		93 (50.5%)	58 (45.3%)	
Occupation							
Farmer	14 (4.5%)	4 (2.5%)	10 (6.6%)	.278	14 (7.6%)	0 (.0%)	<.001
Trader	33 (10.6%)	16 (9.9%)	17 (11.3%)		31 (16.8%)	2 (1.6%)	
Artisan	18 (5.8%)	11 (6.8%)	7 (4.6%)		17 (9.2%)	1 (0.8%)	
Pupil/student	139 (44.6%)	67 (41.6%)	72 (47.7%)		17 (9.2%)	122 (95.3%)	
Civil servant	30 (9.6%)	16 (9.9%)	14 (9.3%)		30 (16.3%)	0 (0.0%)	
Unemployed	21 (6.7%)	11 (6.8%)	10 (6.6%)		19 (10.3%)	2 (1.6%)	
Others	57 (18.3%)	36 (22.4%)	21 (13.9%)		56 (30.4%)	1 (0.8%)	

Abbreviations: FCT, Federal Capital Territory, Nigeria; SAFIAN, Surveillance for Acute Febrile Illness Aetiology in Nigeria.

**Figure 1.**
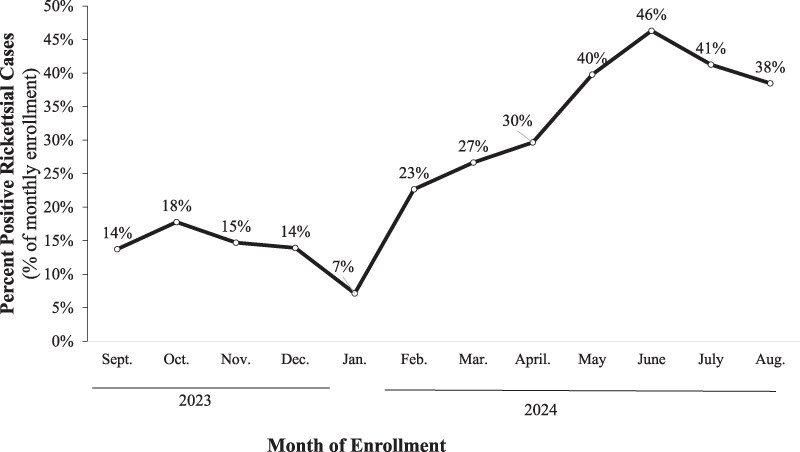
Monthly percent positives of *Rickettsia* among SAFIAN enrollees from September 2023–August 2024. August 2023 and September 2024 data were removed from these figures because enrollment was low for both months (n = 9 and n = 6, respectively). Abbreviations: SAFIAN, Surveillance for Acute Febrile Illness Aetiology in Nigeria.

To understand the seasonal patterns, we calculated monthly prevalence over the course of the study (Figure 1). Monthly prevalence of *Rickettsia* detection varied throughout the study period, with the lowest prevalence observed in January (7.0%) and the peak in June (46.3%).

### Clinical Presentation

In addition to fever, clinical features observed were headache (67.6% of rickettsial-positive patients), nausea and vomiting (28.2%), abdominal pain (19.2%), cough (19.2%), muscle pain (14.4%), joint pain (12.8%), and diarrhea (11.2%)(Table 2). Other, less-common clinical presentations were weakness, runny nose, chest pain, dizziness, loss of appetite, seizure, neck pain, and bleeding. Rash was observed in only 1.9% of rickettsial patients, and none had eschars. Participants in Edo State reported diarrhea, vomiting, and stomach pain (15.9%, 34.4%, and 28.5%) more often than in FCT (6.8%, 22.4%, and 10.6%). Adults experienced joint pain (15.8%) and muscle pain (19.6%) more often than children (8.6% and 7.0%).

**Table 2.** Clinical Characteristics of Enrolled AFI Participants Testing Positive for *Rickettsia* spp.

		Study Sites			Age		
Symptoms of *Rickettsia*	Total (N = 312)	FCT (N = 161)	Edo State (N = 151)	*P*	Adult (N = 184)	Children (N = 128)	*P*
Symptom							
Fever	312 (100.0%)	161 (100.0%)	151 (100.0%)		184 (100.0%)	128 (100.0%)	
Headache	211 (67.6%)	101 (62.7%)	110 (72.8%)	.056	121 (65.8%)	90 (70.3%)	.398
Rash	6 (1.9%)	1 (0.6%)	5 (3.3%)	.084	4 (2.2%)	2 (1.6%)	1.000
Joint pain	40 (12.8%)	20 (12.4%)	20 (13.2%)	.828	29 (15.8%)	11 (8.6%)	.063
Weakness	2 (0.6%)	2 (1.2%)	0 (0.0%)	.499	2 (1.1%)	0 (0.0%)	.515
Cough	60 (19.2%)	41 (25.5%)	19 (12.6%)	.004	31 (16.8%)	29 (22.7%)	.200
Diarrhea	35 (11.2%)	11 (6.8%)	24 (15.9%)	.011	23 (12.5%)	12 (9.4%)	.390
Vomit/nausea	88 (28.2%)	36 (22.4%)	52 (34.4%)	.018	45 (24.5%)	43 (33.6%)	.078
Stomach pain	60 (19.2%)	17 (10.6%)	43 (28.5%)	<.001	33 (17.9%)	27 (21.1%)	.486
Muscle pain	45 (14.4%)	24 (14.9%)	21 (13.9%)	.802	36 (19.6%)	9 (7.0%)	.002
Any other symptoms	7 (2.4%)	1 (0.7%)	6 (4.1%)	.066	5 (3.0%)	2 (1.6%)	.703

Abbreviations: AFI, acute febrile illness; FCT, Federal Capital Territory.

### Risk Factors

The most common risk factor for rickettsial infection was contact with a domestic animal (34.3%) commonly dog, cat, goat, sheep, and pig (Table 2). There were no cases that had contact with a wild animal. Outdoor activity (defined as having spent time in the forest or bush within the past 2 weeks before illness) was reported by 22.8% of the cases. Underlying comorbidities like human immunodeficiency virus infection, diabetes, cancer, hypertension, sickle cell disease, and chronic kidney disease were seen in 17.3% of the cases (Table 3).

**Table 3.** Risk Factors for *Rickettsia* spp. Infection Among SAFIAN Participants (n = 312)

Exposure Information	*Rickettsia* Positive	*P*
Risk factor		
Recent travel	0 (0.0%)	.3037
Animal contact^a^	107 (34.3%)	.5970
Contact with wildlife^b,c^	0 (0.0%)	.1529
Contact with domestic animals^b^	107 (34.3%)	
Outdoor activity^d^	71 (22.8%)	.3097
Comorbidities^e^	54 (17.3%)	.2078

Abbreviation: SAFIAN, Surveillance for Acute Febrile Illness Aetiology in Nigeria.

^a^Contact with at least 1 animal (dog, cat, cow, pig, goat, sheep, chicken, guinea fowl, rodent, bat, monkey, deer, other), regardless of status of animal (alive, dead, sick).

^b^Denominator includes those with no contact with any of the animals that were asked about.

^c^Wildlife includes: rodent, bat, monkey, deer.

^d^Spent time in forest or bush during the 2 weeks before illness.

^e^Comorbidities include: human immunodeficiency virus, diabetes, cancer, hypertension, sickle cell, tuberculosis, seizure, kidney disease, liver disorder, heart disease, other conditions.

### Serological Testing

A total of 28.7% (n = 171) participants tested positive for *Rickettsia* spp. IgG and/or IgM via enzyme-linked immunosorbent assay. Significantly more adults had seroconverted, as compared with children. This overall rate reflects the TAC-based detection rate of *Rickettsia* spp., providing supportive evidence of high prevalence of *Rickettsia* spp. infections in this population (Table 4).

**Table 4.** Serological Positivity of *Rickettsia* spp. Among UATH Enrollees

		Age		
Complications/Mortality of *Rickettsia*	Total (N = 596)^a^	Adult (N = 373)	Children (N = 223)	*P*
Complication				
IgG and/or IgM positive	171 (28.7%)	139 (37.3%)	32 (14.3%)	<.0001
IgG positive	119 (20.0%)	109 (29.2%)	10 (4.5%)	<.0001
IgM positive	66 (11.1%)	44 (11.8%)	22 (9.9%)	.4673

Abbreviations: Ig, immunoglobin; UATH, University of Abuja Teaching Hospital.

^a^Serum samples were not available for 4 UATH enrollees.

### Coinfection With Other Pathogens

Coinfection with other pathogens was found in 36.2% of the cases. The most common coinfection was with *Plasmodium* spp. (25%). Less common coinfections were observed with Lassa fever (8%), *Brucella* spp. (2.9%), Dengue fever virus (0.6%), *Neisseria meningitidis* (0.3%), Zika virus (0.3%), O’nyong’nyong virus (0.3%), *Salmonella* (0.3%), *Coxiella burnetii* (0.3%), Mpox virus (0.3%), and Chikungunya virus (0.3%).

### Outcome

The overall mortality rate was 4.2% among the rickettsial cases, with up to 6.8% mortality in FCT (Table 5). Mortality was significantly higher among the adult cases (6.5%) compared to the pediatric cases (0.8%) (*P* = .013). The renal system was mostly affected in severe cases; acute kidney injury was the most common complication (4.2% of cases). Other complications observed were septic shock (1.6%) and seizures (0.6%). A total of 23.9% of the cases had prolonged hospitalization of greater than 8 days, whereas 7.1% were hospitalized for more than 15 days.

**Table 5.** Complications and Mortality Associated With Rickettsial Infection Among Participants Enrolled in the SAFIAN Study

		Study Sites			Age		
Complications/Mortality of *Rickettsia*	Total (N = 312)	FCT (N = 161)	Edo State (N = 151)	*P*	Adult (N = 184)	Children (N = 128)	*P*
Complication							
Organ failure/issues^a^	0 (0.0%)	0 (0.0%)	0 (0.0%)		0 (0.0%)	0 (0.0%)	
Sepsis	5 (1.6%)	5 (3.1%)	0 (0.0%)	.061	4 (2.2%)	1 (0.8%)	.652
Neurological issues^b^	2 (0.6%)	2 (1.2%)	0 (0.0%)	.499	2 (1.1%)	0 (0.0%)	.515
High blood pressure	10 (3.2%)	10 (6.2%)	0 (0.0%)	.002	10 (5.4%)	0 (0.0%)	.006
Kidney issues^c^	13 (4.2%)	11 (6.8%)	2 (1.3%)	.015	12 (6.5%)	1 (0.8%)	.018
Prolonged hospitalization (8+ days)	74 (23.9%)	48 (30.0%)	26 (17.3%)	.009	47 (25.7%)	27 (21.3%)	.369
Prolonged hospitalization (15+ days)	22 (7.1%)	16 (10.0%)	6 (4.0%)	.040	16 (8.7%)	6 (4.7%)	.175
Mortality/deceased	13 (4.2%)	11 (6.8%)	2 (1.3%)	.015	12 (6.5%)	1 (0.8%)	.013

Abbreviations: SAFIAN, Surveillance for Acute Febrile Illness Aetiology in Nigeria; FCT, Federal Capital Territory.

^a^Includes liver cirrhosis, congenital heart failure.

^b^Includes seizure, neurological care.

^c^Includes dialysis, kidney disease, or diabetes.

## DISCUSSION

This study reports the first detection of rickettsial infection in humans in Nigeria, marking a significant milestone in understanding its epidemiologic and clinical manifestations. Although not intended for frontline diagnosis, these TAC screening results can inform future research, public health response, and clinical decision making for rickettsial infections.

In Nigeria and other countries in sub-Saharan Africa where malaria is endemic, patients presenting with fever are often presumed to have malaria. Across Africa, rickettsial infections are rarely diagnosed, in part, because the disease has a nonspecific clinical presentation, and most clinicians have limited awareness about it. In addition, lack of availability of reliable laboratory tests in healthcare facilities makes it difficult for clinicians to diagnose rickettsiosis.

The SAFIAN study aimed to identify the infectious etiology of AFI among patients presenting at 2 tertiary hospitals in Nigeria. The study detected *Rickettsia* spp. molecularly in 26% of the study participants, and serologically in 28.7%. The high detection rate in this population was unexpected because this pathogen has not been reported in Nigeria in humans; however, literature confirms that similar prevalence rates are common in AFI populations in Asia, Africa, and the Americas. For example, in a similar study in Uganda by Kigozi et al (2023), SFG *Rickettsia* was found in 26.2% and typhus group *Rickettsia* in 7.6% of AFI cases [12]. In India, Mansoor et al (2021) reported that 15.4% of patients that presented to a tertiary care hospital with undifferentiated fever had rickettsial infection [13]. The overall seroprevalence of rickettsial infection in 2 rural villages in Senegal was found to be 21.4% and 51% [14].

Seasonal variations followed a trend of increased rickettsial cases during the rainy season (April–October). *Rickettsia* spp. pathogens are spread by ticks (*R*. *africae*, *R. conorii*) and fleas (*R. typhi*, *R. felis).* In Nigeria, the peak season for tick and flea activity occurs during the wet season (March–October) [15]. Tick abundance has also been shown to increase after the onset of the first scattered rains. Accordingly, the rise and peak of *Rickettsial* spp. detections among SAFIAN participants happened during these months in Nigeria [16]. However, as noted by Oguntomole et al (2018), although research on mosquitos is abundant, there is a paucity of data about pathogens found in association with ticks and fleas, such as *Rickettsia* spp [15]. While Rickettsia had not been detected in humans in Nigeria previously, it had been detected in ticks collected from livestock (*R. africae* and *R. massiliae*) [17, 18]. Accordingly, it was determined that *Rickettsia* spp. had a transmission potential to humans in Nigeria [19].

More men than women were affected at ISTH, located in a rural community compared to UATH in an urban setting. Similar findings were reported in India and Tunisia, with male preponderance among rural dwellers compared to urban dwellers [13, 20]. Many men in rural communities engage in farm work and other outdoor activities that expose them to tick vectors.

Typical clinical symptoms of rickettsial diseases range from mild to severe, and often include fever, headache, malaise, and arthralgia [21–23]. The SAFIAN study found similar clinical features in this febrile population, with headaches, nausea and vomiting, abdominal pain, cough, muscle pain, and joint pain as commonly reported symptoms. Many rickettsioses present with a maculopapular, vesicular, or petechial rash or sometimes an eschar at the site of the tick bite. Some studies have reported that the presence of a rash is a major clinical finding. In a study of rickettsial fever among children in India, Kusagur et al (2022) reported the presence of a maculopapular rash in 57.8% of the children [24]. Among adults with rickettsial disease in Tunisia, 86.4% of them presented with a rash [25]. A literature review of Rocky Mountain spotted fever in the United States showed that 80% of the cases had a rash [26]. Contrary to these findings, only 1.9% of the participants in this study had a rash and no patient had an eschar. Generally, it is more challenging to identify rashes among Africans with darker skin tones because redness and other common signs of inflammation may not be as easily visible as they are in lighter skin. This may explain why fewer participants reported a rash in this study.

Close contact with domestic animals, such as dogs, pigs, goats, and sheep was the major risk factor reported by 34.3% of the participants in this study. From central Tunisia, Kaabia et al (2009) reported that 94.5% of hospitalized patients with rickettsiosis had contact with a domestic animal [25], whereas Mouffok et al (2006) reported that 90% of patients with a clinical diagnosis of Mediterranean spotted fever in Algeria were exposed to dogs [27]. This is not an unexpected finding as several authors working with animals in Nigeria and other countries have isolated pathogenic *Rickettsia* from domestic and wild animals [11, 13, 28, 29]. Therefore, close contacts with these animals poses a major risk of being infected with *Rickettsia*. Among the 40.2 million agricultural households in Nigeria, 48% practice livestock farming, mainly rearing of poultry, goats, cattle, pigs, and sheep [30]. This places a substantial proportion of the population at risk of rickettsial infection.

Rickettsial infections can have a range of outcomes, from complete recovery without sequelae to severe complications and death. Untreated or delayed treatment can lead to complications and death. Although our TAC assay did not speciate *Rickettsia*, serology indicated SFG presence, which includes species ranging from mild/moderate to severe illness, such as *R. africae* and *R. conorii* [31]. In this study, 95.8% of the patients who tested positive for *Rickettsia* spp. with polymerase chain reaction–diagnosed rickettsial infections recovered fully and were discharged, whereas 4.2% had complications and died. Mortality rates of 0% and 3.2% were reported in Tunisia and Algeria, respectively [25, 27]. Although some of the patients in the SAFIAN study received antibiotics during the period of hospitalization, none of them received anti-rickettsial agents doxycycline and chloramphenicol. This could explain the relatively higher mortality rate in this study. The complications encountered were acute kidney injury, septic shock, and seizures, which is consistent with reports from other studies [32–34]. Rickettsial infections cause vasculitis, which may be widespread with multiorgan involvement in severe cases. The mortality rate among the pediatric cases was 0.8%, which was significantly lower when compared to the mortality among adults. This finding concurs with the report of Stewart et al (2020) that showed that pediatric rickettsial infections have a relatively benign clinical course in tropical Australia [35].

Our findings have important implications for clinical management. Despite more than one quarter of patients testing positive for *Rickettsia* spp., none received anti-rickettsial therapy. In malaria-endemic, resource-limited settings where rickettsial diagnostics are largely unavailable, empirical therapy is an important consideration. Doxycycline, which is effective against a broad range of rickettsial pathogens and some malaria species, represents a potentially valuable empirical option for patients with acute undifferentiated fever. The advantages of this approach include low cost, wide availability, and coverage of multiple likely etiologies; however, drawbacks include the risk of inappropriate use in non-rickettsial, non-malarial febrile illnesses, potential adverse effects, and broader concerns about antimicrobial stewardship. While strengthening diagnostic capacity is the goal, in the interim, the judicious incorporation of doxycycline into clinical algorithms for undifferentiated fever should be carefully evaluated through operational research.

A notable proportion of patients who tested positive for *Rickettsia* spp. also had evidence of *Plasmodium* infection. Because the TAC assay detected *Plasmodium* at the genus level, we could not resolve species, although *P. falciparum* is likely the predominant species in Nigeria. In malaria-endemic settings, asymptomatic parasitemia is common, making it difficult to distinguish between incidental detection and true concurrent infection. Given the overlapping, nonspecific clinical features of malaria and rickettsioses, some of the febrile presentations attributed to 1 pathogen may, in fact, be due to the other, or to both in combination. These findings highlight both the diagnostic challenges posed by coinfections and the importance of integrating more specific diagnostic approaches to clarify the relative contributions of malaria and rickettsial infections to febrile illness in sub-Saharan Africa [36].

This study has several limitations. First, detection of *Rickettsia* spp. was based on results from a TAC screening tool; accordingly, results cannot be interpreted as diagnostic results and require confirmatory testing to be actionable. Although we did not conduct confirmatory testing of TAC results, the serological findings bolster the validity of our TAC results. Second, species-level identification of *Rickettsia* was not performed, precluding inferences about clinical variability across species. Third, the hospital-based design may overrepresent more severe cases and does not reflect community-level prevalence. Fourth, treatment data were incomplete; although patients received antibiotics, no targeted anti-rickettsial therapy was administered. Finally, absence of follow-up after discharge limits evaluation of long-term outcomes. These limitations also identify opportunities for further investigations.

## CONCLUSION

In Nigeria, the burden of rickettsial diseases in humans has not been previously reported. This study found a high prevalence of 26% of rickettsial infection via a molecular screening assay. These results are not confirmed as they were generated from a research-use only assay; however, the body of evidence suggests the presence of rickettsiosis, including comparable serological positivity rates, the commonalities of symptoms and seasonal trends with other epidemiological reports of rickettsiosis.

The presence of a rash or eschar is not a common clinical presentation of rickettsial disease in Nigeria. The mortality rate is high in the absence of appropriate antibiotics. These findings highlight the need for additional surveillance studies of *Rickettsia* spp. in Nigeria that incorporate confirmatory testing, clinical diagnostics, speciation, and the development of clinical profiles and improved characterization of underdiagnosed rickettsial fevers in Nigeria. Clinicians should, therefore, suspect and test for rickettsial infection in patients presenting with undifferentiated fever.
